# Potential theranostics of circulating tumor cells and tumor-derived exosomes application in colorectal cancer

**DOI:** 10.1186/s12935-020-01389-3

**Published:** 2020-07-06

**Authors:** Somayeh Vafaei, Raheleh Roudi, Zahra Madjd, Amir Reza Aref, Marzieh Ebrahimi

**Affiliations:** 1 Oncopathology Research Center, Iran University of Medical Sciences (IUMS), Hemmat Street (Highway), Next to Milad Tower, Tehran, Iran; 2 Department of Molecular Medicine, Faculty of Advanced Technologies in Medicine, Iran University of Medical Sciences, Tehran, Iran; 3 Department of Stem Cells and Developmental Biology, Cell Science Research Center, Royan Institute for Stem Cell Biology and Technology, ACECR, Tehran, Iran; 4 Department of Medical Oncology, Dana-Farber Cancer Institute, Harvard Medical School, Boston, USA

**Keywords:** Colorectal cancer, Circulating tumor cells (CTCs), Tumor-derived exosomes (TDEs), Clinical trial, Theranostic

## Abstract

**Background:**

At the present time, colorectal cancer (CRC) is still known as a disease with a high mortality rate. Theranostics are flawless scenarios that link diagnosis with therapy, including precision medicine as a critical platform that relies on the development of biomarkers particularly “liquid biopsy”. Circulating tumor cells (CTCs) and tumor-derived exosomes (TDEs) in a liquid biopsy approach are of substantial importance in comparison with traditional ones, which cannot generally be performed to determine the dynamics of the tumor due to its wide restriction of range. Thus, recent attempts has shifted towards minimally noninvasive methods.

**Main text:**

CTCs and TDEs, as significant signals emitted from the tumor microenvironment, which are also detectable in the blood, prove themselves to be promising novel biomarkers for cancer diagnosis, prognosis, and treatment response prediction. The therapeutic potential of them is still limited, and studies are at its infancy. One of the major challenges for the implementation of CTCs and TDEs which are new trends in translational medicine is the development of isolation and characterization; a standardizable approach. This review highlights and discusses the current challenges to find the bio fluids application in CRC early detection and clinical management.

**Conclusion:**

Taken together, CTCs and TDEs as silent drivers of metastasis can serve in the management of cancer patient treatment and it is of the upmost importance to expand our insight into this subject. However, due to the limited data available from clinical trials, further validations are required before addressing their putative application in oncology.
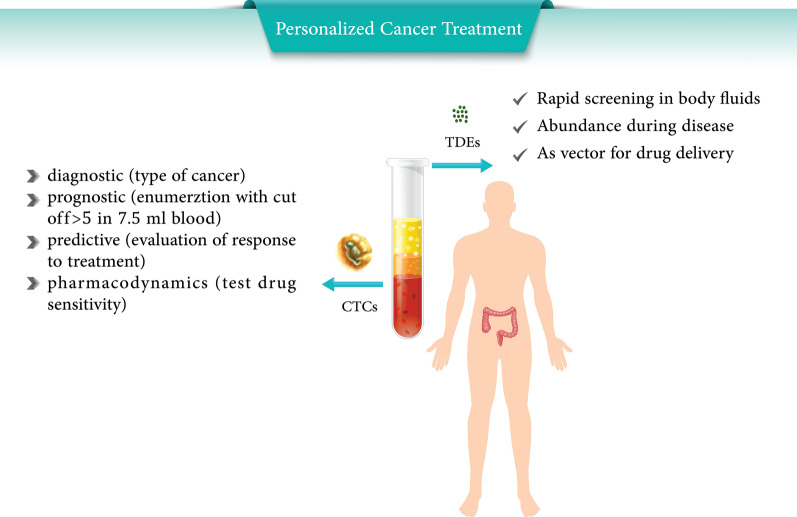

## Background

Colorectal cancer (CRC) is the third leading cause of cancer-related mortality and morbidity [1] and fifty percent of patients suffering from metastasis undergo surgery [2] which creates huge obstacles in treatment and eventually leads to patient death. Unfortunately, primary tumor resection appears not able to evacuate seeded malignant growth cells, and guides dormant cancer cells to induce metastatic growth leading to recurrence by circulating tumor cells (CTCs) and tumor-derived exosomes (TDE) in some cases [3]. Traditional biomarkers (CEA, CA19-9 and FOBT), as well as colon/sigmoidoscopy play an unsatisfactory specificity roles in colorectal screening [4]. Since the demerits of these various CRC screening tests are considerable [5]; shifting to repeatable noninvasive methods such as liquid biopsy attracted much attention [6, 7].

CTCs and TDEs are liquid biopsy tools which can provide complementary information about the whole tumor [8, 9]. Detection of them as a source of molecular markers (DNA, RNA, miRNA and proteins) provide relevant predictive gene signatures. They can be isolated from body fluids to elucidate patient’s clinical guidance and mediated tumor signatures [10, 11]. They are important in diagnostic, prognostic and cancer staging and has profitable usage in the estimation of relapse risk, therapeutic targets identification, intervention for stratification, sequential and continuous checking of treatments, determination of predictive information, and minimal residual disease follow up [12, 13]. Standardization of integrated pre/post analytical workflows of sample handling (isolation and characterization) must be greatly considered as priorities in increasing patient survival due to accurate therapy decision making [14]. The current review summarizes clinical translation, isolation methods, and crosstalk of CTCs and TDEs as a practical concept in colorectal cancer liquid biopsy.

## CTCs & TDEs in CRC

### Comprehensive concept and biology

The main step in cancer progression is detachment, invasion of cancer cells and extravasation in order to metastasize to survive [15]. The most important materials shed into the systemic blood to establish pre-metastatic niche in maintenance of stemness and promote immune evasion include CTCs, TDEs and even cancer stem cells (CSCs). CTCs as a valuable disease indicator [16] among thousands of tumor cells leak into circulation and can survive. This ability is due to various mechanisms attributed to it such as resistance to blood shearing forces, anoikis, immune system attack and also down regulation of *c*-*myc, β*-*catenin* and *Ki*-*67*, and over expression of *CD47* [17]. An average number of CTCs in a metastatic patient is between 5 and 50 in 7.5 cc peripheral blood, thus it is extremely low and suffers a number of challenges such as high fragility, low half-life, gain/loss of cell markers, vast range of phenotypic and genotypic heterogeneity, and plasticity [18].

On the other hand, the concept of CSCs as a small population with diverse phenotype, self-renewal ability, cellular differentiation and resistance to conventional therapies can contribute to tumor progression [19, 20]. Self- homing CTCs have been reported as delivery vehicles for anti-cancer therapeutics. Hence, detection, enumeration and molecular characterization of CTCs and CSCs are considered to be impediment factors in cancer clinics [21].

Tumor cells shed under epithelial mesenchymal transition (EMT) or by centrosome amplification triggering or external forces [22]. In addition, the mesenchymal epithelial transition (MET), as a reverse process, establishes micro metastasis. Advancing knowledge related to dominant drivers in cancer complex interactions is critical for therapeutic scheme design [23].

**Fig. 1:**
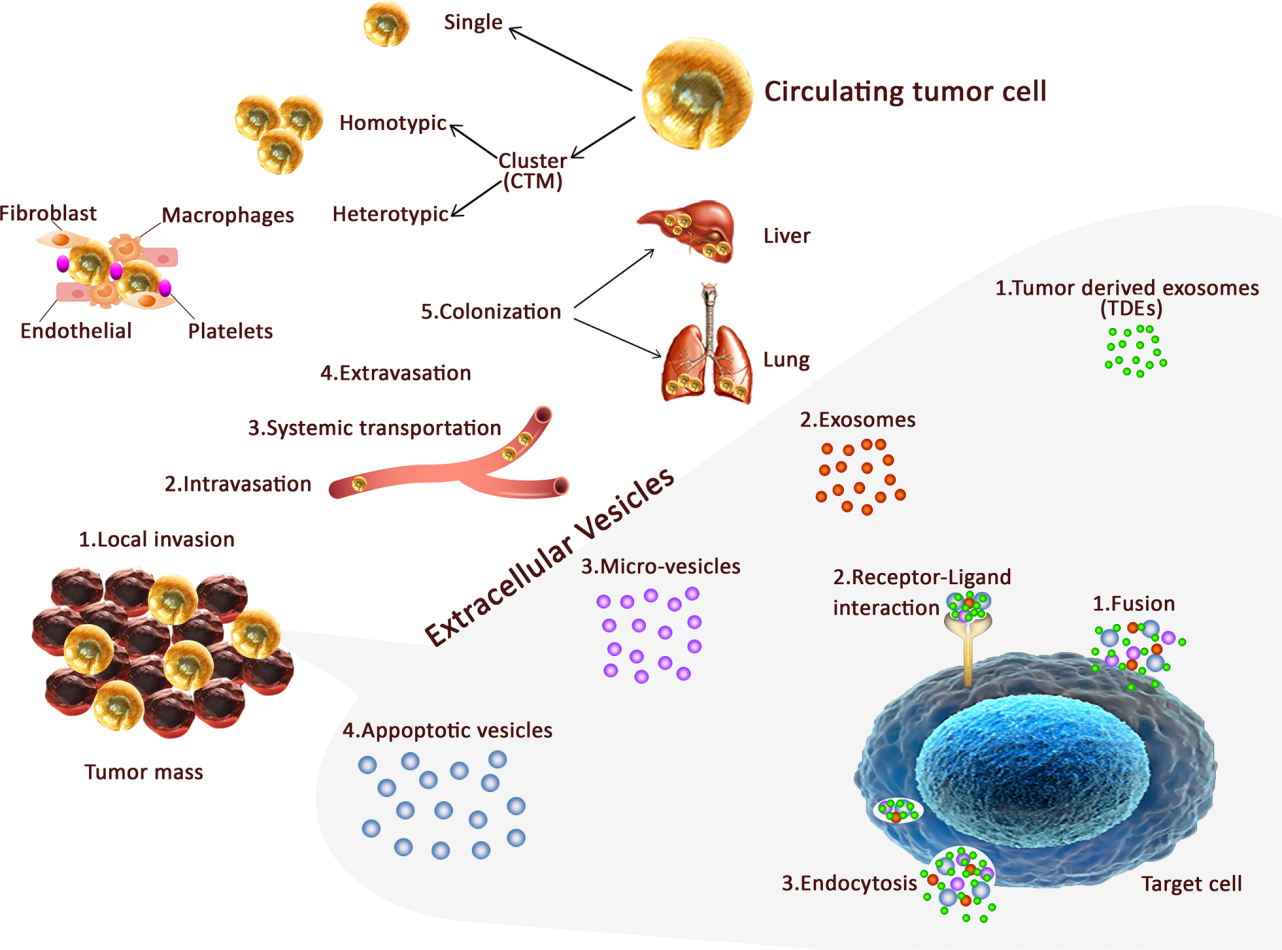
The different types of CTCs and extra vesicles in colorectal cancer patient blood circulation. **a** tumor mass released circulating tumor cells to the blood circulation which intravasate to the blood vessel and via systematic transportation can extravasate and establish a colony in the secondary metastatic body such as liver and lung. CTCs can move in single or cluster ones that are homotypic or can accompany fibroblast, endothelial, platelets and macrophages as heterotypic cells. **b** Extracellular vesicles also can be shed from tumor mass into the next microenvironment that consists of tumor-derived exosomes (TDEs), exosome, microvesicles and apoptotic vesicles that are different from each other in size. These vesicles can be received via fusion, receptor-ligand interaction, and endocytosis by their selective target

CTCs may exist as single cells with a wide range of EMT phenotype or in clusters with platelets, and/or reactivated stromal cells and macrophages [24]. CTC phenotype incorporate with epithelial tumor cells as well as EMT, half-breed (epithelial/EMT), irreversible EMT cancer cells, and CSCs that is shown in Fig. 1 [25]. Platelets surround the CTCs as supporters and promote tumor cells EMT and facilitate development in the distant organs [26]. CTC numbers before and during treatment are an independent indicator of overall survival (OS) and progression-free survival (PFS), by genome, expression, protein and functional analysis [27]. CTCs from 2004 in three metastatic cancers were introduced in clinics as an independent prognostic factor of survival [21].

Additionally, extracellular vesicles (EVs) contain apoptotic bodies (500–1000 nm), microvesicles (100–350 nm), and exosomes (30–150 nm) [28]. Pan et al. in 1983, for the first time, introduced and confirmed exosomes [29, 30] which are vesicles secreted by various kinds of cells and include a broad repertoire of cargo such as DNAs, RNA, proteins and lipids (Fig. 1) [31]. TDEs are originated from multivesicular bodies (MVBs) and the plasma membrane fusion and release their contents to be uptaken by targets. TDEs are capable of modulate cellular activities via transferring genetic data of tumor and reflect the original cell nature. Exosomes which promote adhesion, not only play a significant role in triggering signaling pathways such as immune escape and inflammatory responses, but also act in the diagnosis, prognosis and treatment assessment [21]. Additionally, they have been engineered as vectors in cancer intervention and affect the tumor microenvironment [32]. They modulate the immune response, regulate intercellular communication, mediate tumor resistance by drug efflux, and are even introduced as potential biomarkers in various diseases [33, 34].

### General approaches in isolation and characterization

Considering the importance of these two biomarkers in basic research and clinical translation, investigating the isolation, enrichment, molecular and bioinformatics analysis of them as opposed to a complex biological background is crucial [35]. In the past, scientific proof on CTCs via RT-PCR and immunocytochemistry based on epithelial-specific antibodies gave false positive results [36].

CTC detections include five technical indicators: capturing rate efficiency or recovery, purity in the enriched sample, CTC concentration limitation in the blood, throughput and biocompatibility [37]. Three general mechanisms of CTC enrichment have been developed based on the importance of isolation approach namely: (1) biological, (2) physical and (3) functional, which have been illustrated in Table 1. (1) Immuno/magnetic affinity surface/intra cellular marker based on (peptide/aptamer/antibodies) affinity [38]: (1-A) In positive selection/capture, CTCs are directly isolated. The first and gold standard systems worked based on *EpCAM* named CellSearch™ as the only FDA platform in which labeling with an avidin–biotin anti-*EpCAM*-ferrofluid complex was employed; [39] this method can also be used in vivo assay [40]. (1-B) negative selection can be helpful for avoiding selection bias marker based on tumor heterogeneity via depletion of abundant leucocytes through removal *CD45* and other antigens. (1-C) combination of both selection such as Liquid Biopsy platform [41].

**Table 1:** Enrichment/isolation approaches of CTCs based on the inherent characteristics

Total approach	Methods/kits
Biological (Immuno-affinity)	
Negative selection	Rosettesep [50]/Easysep [51]/Magnetic-activated cell sorting (MACS) [52]/Fluorescence-activated cell sorting (FACS) [53]/Dynal Invitrogen [54]/CTC-iChip [55]/Ephesia [56]/GEDI [57]/QMS [58]
Positive selection	Cell search [39]/Magnetic-activated cell sorting (MACS) [52]/Fluorescence-activated cell sorting (FACS) [53]/Epic system [59] Magsweeper [60]/Rosettesep/Easysep/Cytoquest/Adnatest [61]/GILUPI Nanodetector [62]/Liquid Biopsy (Cynvenio) [45]/Dynal Invitrogen
Physical	
Size	Label-free/Spiral/Vortex/Microfiltration/Vycap/IsoFlux (Fluxion) [62]/Rare cell Devices Isolation by SizE of Tumor/Trophoblastic Cells (ISET) [63]/DEPArray [64]/(Silicon Biosystems)/ApoStream (ApoCell) [65]/Clear cell Parsortix [66]/Flexible micro spring array (FMSA) [67]/fiber-optic array scanning technology (FAST) [68]/Metacell [69]**/**Resettable Cell Trap/CellSieve/FaCTCheckr/ScreenCell/ClearCell FX [70]
Gradient density	OncoQuick (Grenier Bio-One) Ficoll-Paque [71]/Rosettesep/CyteSealer/AccuCyte [72]
Di-electrophoretic (DEP)	DEP-FFF/LFFF-DEP [73, 74]
Functional analysis	EPISOT/Vita-Assay (Vitatex) [75]**/**Epithelial ImmunoSPOT [38]/in vivo photoacoustic (PA) flow cytometry (PAFC) [62]

(2) Physical/direct enrichment of CTCs (e.g. size and deformability, gradient density and di-electrophoresis) are the second criteria that can be used to enrich cancer cells from blood cells positively and/or negatively. CTCs are bigger than 12 µm in comparison with Lymphocytes and neutrophils which are lower than 12 µm [42].

(3) Functional measurement exploit CTC cellular activity, enrichment and separation, namely epithelial immunospot secreted tumor-marker proteins, and have been reported in several cancers [43].

Microfluidics has opened a new window in general methods via hydrodynamics/inertial focusing/spiral to separate CTCs from other blood cells passively. Utilizing immobilized specific CTC antibodies on microchips/micro-posts or in a herringbone design improve cell viability and efficiency [44]. Miniaturization of the traditional laboratory instrument followed by in situ cells capturing, sorting and analyzing have attracted much attention such as CTC-chip [45], graphene oxide–go chip [46], hb-chip [47], gem chip [48].

All of these abovementioned methods require identity confirmation of the captured, associated cells with differential staining using high resolution imaging with DAPI (nucleated cells), *CK* (*CK20*, *CK19*, *CK18*, and *CK8*) (epithelial structural), and anti-*CD45* (CTCs) as *DAPI*+*/CK*+*/CD45*− from circulating white blood cells (WBCs). The time for detection of CTCs must be done at least 7 days postoperatively and also the whole CTC operation process had a significant impact on CTC results and must be carried out quickly [18, 49].

TDE isolation and purification among a mixture of EVs are technically unavailable at the moment. Therefore, novel isolation methods are crucial to enrich the specific subtypes [76]. Three general approaches for exosome isolation were summarized in Table 2 based on: (1) Physical characters including size and gradient density centrifugation (DGC) and ultracentrifugation (UC) (increasing centrifugal force ≥ 100,000*g*) apply to progressively eradicate unwanted smaller debris and bigger subpopulations of vesicles as a gold standard [77]. Furthermore, filtration and size exclusion chromatography (SEC) were considered as an important approach in this category. UC is a labor intensive and time-consuming procedure that requires specialist laboratory equipment that can be combined with the other modalities such as sucrose gradient and poly ethylene glycol (PEG) to increase the yield [78].

**Table 2:** Enrichment/isolation approaches of exosomes based on the inherent characteristics

	Methods/kits
Physical	Ultracentrifugation [84–87]/Sucrose gradient [88]/Membrane-based filtration/Filter-based/Column-based/Chromatography [89]/Nanowire trapping [90]
Chemical	Exoquick [91]/Exospin/qEV [92]
Biological (Immuno-affinity)	Magcapture™ Exosome isolation kit [93]/Dynabeads^®^/Fluorescence/colorimetric [94]

(2) Chemical properties, samples incubated with a PEG based on their solubility and exosomes separate centrifugation or filtration [79]. Currently, several exosome precipitation kits such as ExoQuick™, Exospin and the other kits are commercially available [80].

(3) Immunoplate- and immunobead-based affinity isolation can be accompanied by performing molecular labeling of the exosome, including *CD81*, *CD9*, *CD63*, *TSG101*, *HSP 70* and *Alix.* Magcapture™ exosome isolation kit PS and *CD63* dynabeads^®^ beads work based on this approach. An ELISA-based method was also developed for exosome detection, in support of functionalized approach via specific antibodies. Characterization of exosomes based on morphology via scanning electron microscope (SEM) and transmission electron microscopy (TEM) can be determined. Then nanoparticle tracking assay (NTA) and dynamic light scattering (DLS) verify wanted vesicle size samples. Finally, their molecular profiling can be defined through conventional ELISA, PCR and western blotting [81, 82].

Alternatively, microfluidic based exochips and poly dimethyl siloxane (PDMS) innovative sorting platform devices by electromagnetic and electrophoretic manipulations have been developed to isolate exosomes. This technology has many advantages such as being user friendly, with quantitative readouts, high sensitivity, is economic, fast and requires minimal sample handling [83].

### Molecular markers

Colorectal cancer has two types including sporadic and hereditary, the first of the two (65%) [95] is directly impressed by personal life-style and the second one consists of familial adenomatous polyposis (*FAP*), due to Adenomatous polyposis coli (*APC*) gene mutations, and *HNPCC*/lynch syndrome, that is caused by *MMR* genes [96].

Colorectal CTC markers included carcinoembryonic antigen (*CEA/CEACAM5*, *7*), *EpCAM*, *CK19* and *CK20* [97, 98]. Colon stem-like cells express *CD44*, *CD166* (*ALCAM*), *CD133* (Prominin-1), *CD29*, *CD24*, *EPCAM*, doublecortin like kinase 1 (*DCLK1*), Leucine-rich repeat-containing G protein-coupled receptor 5 (*Lgr5*) [99, 100]. Additionally, there are some known markers in targeted therapy which have been discussed clinically including *EGFR*, *VEGF, IGF*-*IR* the insulin-like growth factor 1 receptor (IGF-1R), interleukin-4 (*IL*-*4*) and bone morphogenetic protein 4 (*BMP*-*4*) [101].

**Fig. 2:**
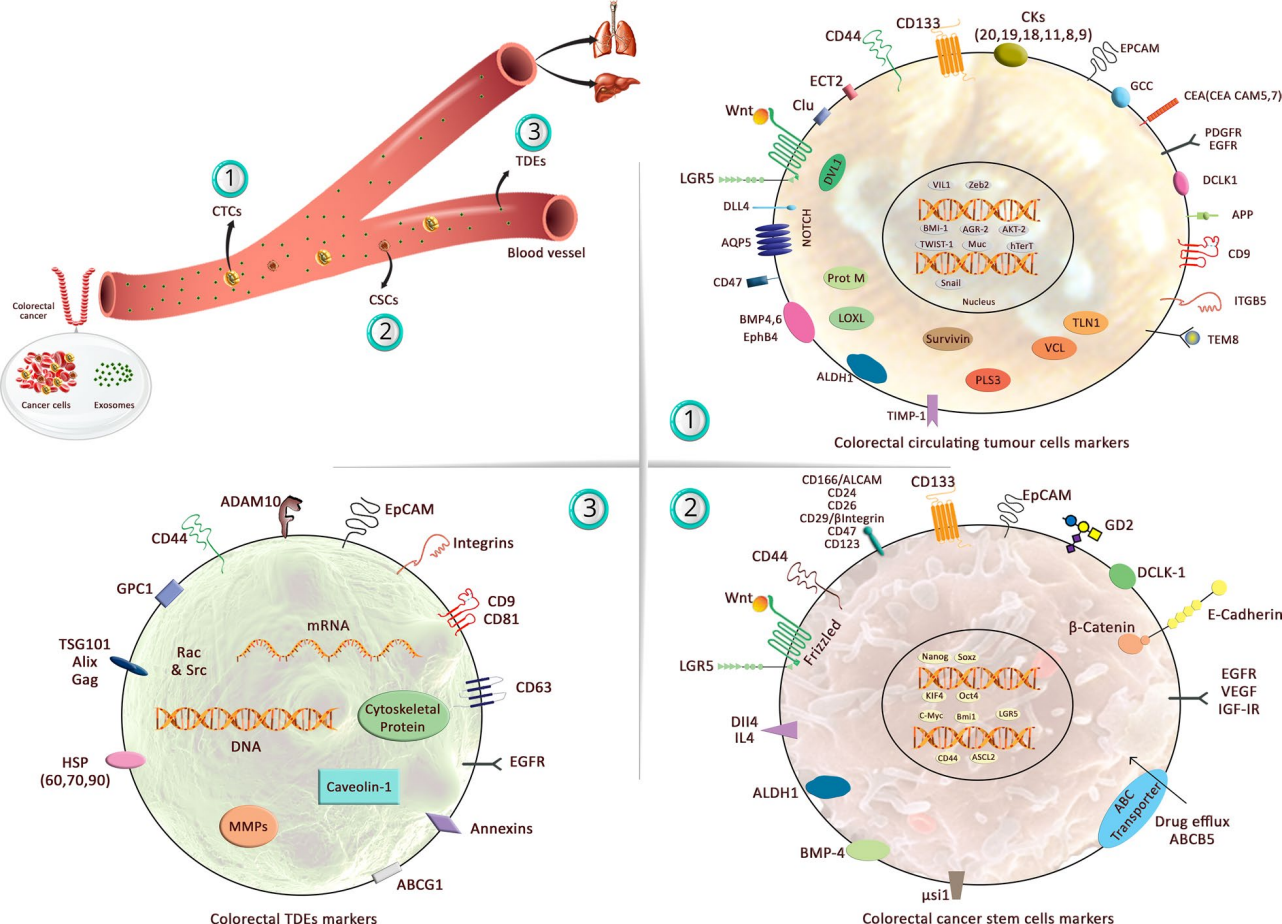
A brief graphical explanation is provided regarding molecular markers expressed in CTCs, CSCs, and TDEs in CRC

Analysis of exosome composition indicated that they express tetraspanins, a class of membrane proteins including *CD9*, *CD63* and *CD81* [102]. The other frequent exosomal proteins are *EpCAM*, *Alix*, and *TSG101* [103], *GTPases*, cytoskeletal proteins, *annexines*, the heat shock proteins (*Hsp70* and *Hsp90*) [104] and integrins [105, 106], of which all of the valuable biomarkers were drawn in Fig. 2.

### Clinical applications to manage patients

CTCs were captured via all the aforementioned approaches that have been discussed and cultured in vivo/vitro named patient-derived xenografts (PDXs) and CTC-derived xenografts (CDXs) although the establishment of permanent CTC lines is very challengeable [21, 107].

In this section, clinical studies concerned with the colorectal CTCs will be mentioned; 63 trials were registered in https://clinicaltrials.gov of which 22 of them were completed and summarized in Table 3. Meta-analyses and large-scale clinical trials declare that patients with CTC number ≥ 5 (per 7.5 ml) were classified as being in the aggressive stage IV and would develop distant metastasis. Meanwhile, CTC level < 3 cells can also be correlated with unfavorable prognostic factor [108] with shorter median OS and PFS [109]. Thus, it can be a vital factor in cancer progression risk assessment and patients must be stratified to be treated promptly based on molecular subtypes [110, 111]. Therefore, higher numbers of CTCs are seen in patients with a greater number of metastatic sites [112]. Regardless of the metastatic site, CTC enumeration (cell-based assays) are sufficient enough as a proper cancer monitoring index whenever *CEA* and other markers levels are not measurable [113]. It is worthy to mention that an elevated CTC number was not necessarily associated with apoptotic CTCs or CTC debris and could be used to interrogate metastatic in patients and contribute to run tumor-associated events [114, 115].

**Table 3:** The complete clinical trials of circulating tumor cells (CTCs) in colorectal cancer

Clinical trials.gov identifier/(refs.)	Investigator, country/year	Condition/patient no.	Methods	Short description
NCT02450422/The detection of circulating tumor cells (CTCs) in patients with CRC undergoing cryosurgery combined with DC-CIK treatment	Wang, China/2013–2015	II–IV/60	Flow cytometry RT-PCR	Test CTCs from patients received cryosurgery and/or DC-CIK treatment, 1 day before and 2 days after
NCT01640444/Influence of BRAF and PIK3K status in patients with RAS wild-type metastatic colorectal carcinoma and < 3 CTC (VISNU-2)	Díaz-Rubio, Aranda, Sastre, Spain/2012–2018	Metastatic/240	CTC count	Influence of BRAF and PIK3K status on the efficacy of FOLFIRI + Bevacizumab or Cetuximab
NCT01163305/PET-CT and CTCs in CRC	Brigette, Hong Kong/2010–2017	Metastatic/84	PETScan, RECIST Criteria	Assessing Chemotherapy (oxaliplatin or irinotecan) response (measuring tumor metabolic)
NCT01943500/Collection of blood specimens for CTC analysis	Sanz-Altamira, USA/2012–2017	II–IV/14	CTC count	Test the sensitivity of a proprietary filtration device designed to capture and concentrate CTCs
NCT03337347/Clinical significance of detecting CEA and CK20 mRNA-positive cells in CRC patients	Duda, Czech Republic/2004–2017	I–IV/256	CTC count RT-PCR	Determine the correlations of CTC in the blood and bone marrow of CRC patients with CEA and CK20 mRNA-positive cells as a negative prognostic factor
NCT01628328/Colonic stent and tumor cell dissemination	Poon, Hong kong/2010–2012	II–IV/40	FACS	Assess impact of metallic stent insertion for obstructing measuring the level of CTCs before and after colonoscopic stenting vs colonoscopy
NCT01722903/Detection of CTCs in patients undergoing surgery for stage IV CRC	Kaifi, USA/2012–2015	Metastatic/26	FMSA device Cell search	Detection of CTCs during CRC syn- and metachronous liver and lung metastases
NCT01212510/Study of circulating markers in serum of patients treated for metastatic CRC (Coca-Colon)	Michel and Rouen, France/2010–2016	Metastasis/200	CTC count Real-time RT-PCR	Measure of tumor markers (blood rate of ACE, CA19-9, CTC, ctDNA)
NCT00351572/Frequency of CTCs in stage II and stage III colon cancer patients	Sawyer, Canada/2006–2006	II–III/30	Cell search	Detect of CTC in patients who have had surgery for CRC presence and recurrence
NCT01640405/Study of first line treatment of patients with metastatic CRC not previously treated and with three or more CTC (VISNU-1)	Díaz-Rubi and Aranda and Sastre, Spain/2012–2018	Metastasis/350	CTC count	To evaluate FOLFOX + bevacizumab versus FOLFOXIRI + bevacizumab as first line treatment of patients with metastatic CRC not previously treated and with three or more CTCs Determine the Correlation of RAS, BRAF and PI3K mutations and clinical anti-tumor activity outcome (PFS, OS, RR)
NCT02029326/Biomarker analysis in metastatic colorectal cancer treated with cetuximab	Samsung Medical Center, Korea/2013–2017	Metastasis/30	Onco dX assay	To analyze expression and activation status of receptor tyrosine kinases in signal transduction pathways in FNA samples and CTCs and identify negative predictive markers to cetuximab and analyze correlation between the quantity of CTCs and treatment response to cetuximab
NCT03640572/Disseminated tumor cells (DTC) in left sided colorectal cancer (LSCC)	Antoni Szczapanik, Assoc, Poland/2018–2019	Metastasis/91	Bone marrow analysis	The incidence of DTC was not related to the depth of infiltration (T feature) being similar in T1–2 and T4 patients There was no statistically significant difference between the incidence of DTC in N− and N+ patients. The 5 years survival rate for the DTC patients was 59, 5% while for the DTC negative patients was 53%
NCT02186236/Detection of oncogenic tumor mutations in the urine and blood of lung and colorectal cancer patients	Memorial Sloan Kettering Cancer Center, USA/2014–2019	IV/84	Molecular analyses	Determine the presence of EGFR mutation in CTC and in cfDNA or RAS/RAF mutation by urine or plasma-based assay as compared to the gold standard of tumor tissue
NCT03008499/High-activity natural killer immunotherapy for small metastases of colorectal cancer	Fuda Cancer Hospital, Guangzhou, China/2016–2019	Patient refuses standard therapies after cancer recurrence/20	–	Determine the safety and the short and long term efficacy of high-activity natural killer cells that evaluated according to local relief degree, PFS and OS
NCT03357276/Mix vaccine for metastatic colorectal cancer	Fuda Cancer Hospital, Guangzhou, China/2016–2019	Patient refuses standard therapies after cancer recurrence/30	–	Determine the safety and the short and long term efficacy of mix vaccine that evaluated according to local relief degree, PFS and OS
NCT03031691/A study of brontictuzumab with chemotherapy for subjects with previously treated metastatic colorectal cancer	OncoMed Pharmaceuticals, Inc, USA/2017–2019	Metastasis/7	–	Determine the safety and pharmacodynamics of brontictuzumab in combination with chemotherapy for subjects with previously treated metastatic CRC Meanwhile, patients went under screening period during treatment period and a post-treatment follow up period in which patients will be followed for survival
NCT02080650/Characterization of circulating tumor cells captured by c-MET (CTC-MET)	Andrew J Armstrong, USA/2014–2017	Metastasis/62	Mesenchymal-marker based ferrofluid (c-MET) and Epithelial cell adhesion molecule (EpCAM) ferrofluid	Determine whether CTCs can be captured using the cMET based ferrofluid Describe the detection rates of both the c-MET CTC capture and the EpCAM CTC capture techniques in each patient
NCT00924092/An open label phase I Study to eval the safety and tolerability of a vaccine (GI-6207) consisting of whole, heat-killed recombinant saccharomyces cerevisiae (yeast) genetically modified to express cea protein in adults with metastatic CEA-expressing	Ravi A Madan, M.D. USA/2009–2019	Metastasis/25	Molecular analyses	Determine the safety and tolerability of escalating doses of a heated-killed yeast-based vaccine that targets tumors that express CEA Evaluate CD4 and CD8 immunologic response to yeast antigen. To evaluate evidence of clinical benefit such as PFS, OR and CTCs decreasing via assessment of tumor markers
NCT00560560/Study using CP-751,871 in patients with stage iv colorectal cancer that has not responded to previous anti-cancer treatments	Pfizer CT.gov Call Center Pfizer, USA, Spain and United Kingdom/2007–2013	IV/168	CTC count	This study will test if there is any survival benefit in patients with refractory metastatic colorectal cancer that receive CP-751,871
NCT00483080/Study of NGR-hTNF as single agent in patients affected by colorectal cancer (CRC)	MolMed S.p.A. Italy/2006–2013	Metastasis/46	–	Evaluation of the safetly of NGR-hTNF on patients who previously treated with fluoropyrimidine, oxaliplatin and irinotecan based regimens and correlation with survival
NCT00335595/Study of bevacizumab alone or combined with capecitabine and oxaliplatin as support therapy in metastatic colorectal cancer patients	Enrique Aranda, M.D.; ph.D., Eduardo Díaz-Rubio, M.D.; ph.D. and Spanish Cooperative Group for Gastrointestinal Tumor Therapy (TTD), Spain/2006–2013	Metastasis/480	CTC count	Compare the free time to disease progression of combination therapy with capecitabine, oxaliplatin and bevacizumab until disease progression versus capecitabine, oxaliplatin and bevacizumab for 6 cycles followed by bevacizumab until disease progression or a premature drop out of the study
NCT02020291/Phase I study to evaluate safety, tolerability, anti-tumour activity and pk profiles of foxy-5 in metastatic breast, colon or prostate cancer	WntResearch AB, Denmark/2013–2016	Metastasis/31	CTC count	Develop Foxy-5 as a first in class anti-metastatic cancer drug via inhibition the development of metastasis by reducing the motility of cancer cells and increasing the survival rates of patients

*RR* response rate, *PFS* progress free survival, *OS* overall survival

In another site, only five clinical trials using the key word ‘colorectal exosome’ were registered that none of them completed. Recently, TDEs have been introduced as promising drug delivery vehicles in targeting different organs and their selective cargo must be determined to increase therapy effectiveness. Thus, scientists are focusing on TDEs components [116] even in inducing anti-tumor immune responses as cancer vaccine candidates [117]. The plasma TDE cargo is enriched in immunosuppressive and immunostimulatory receptor/ligands, MHC molecules and various tumor-associated antigens (TAAs). Their content depends on cellular origin variety and carries oncogenic DNA, microRNAs, proteins and mRNAs [118] such as GPC1^+^, tumor suppressor-activated pathway 6 (*TSAP6*) [119], *ΔNp73* [120], metastatic factors (*TNC*, *MET*, *S100A9*, *S100A8*), signal transduction molecules (*EFNB2*, *JAG1*, *SRC*, *TNIK*), and lipid raft associated components (*PROM1*, *CAV1*, *FLOT1* and *2*). Ji et al. reported *Let*-*7a*-*3p*, *let*-*7f*-*1*-*3p*, *miR*-*574*-*5p*, *miR*-*451a*, *miR*-*7641*, and *miR*-*4454* are common to all EV subtypes [121]. In addition to the detection and co-localization of protein complexes in CRC exosomes, regulation of signaling pathways such as Wnt and *EGFR* ligand, besides autocrine, paracrine, and juxtacrine, contribute in priming of the metastatic niche [122]. Furthermore, inhibition of exosome secretion, besides targeting CSCs, as a new therapeutic strategy, can block tumor associated secretion before chemotherapy [123, 124] and facilitate cross talk between stromal cells and tumors in cancer microenvironment [125].

### Crosstalk in tumor microenvironment (TME)

**Fig. 3:**
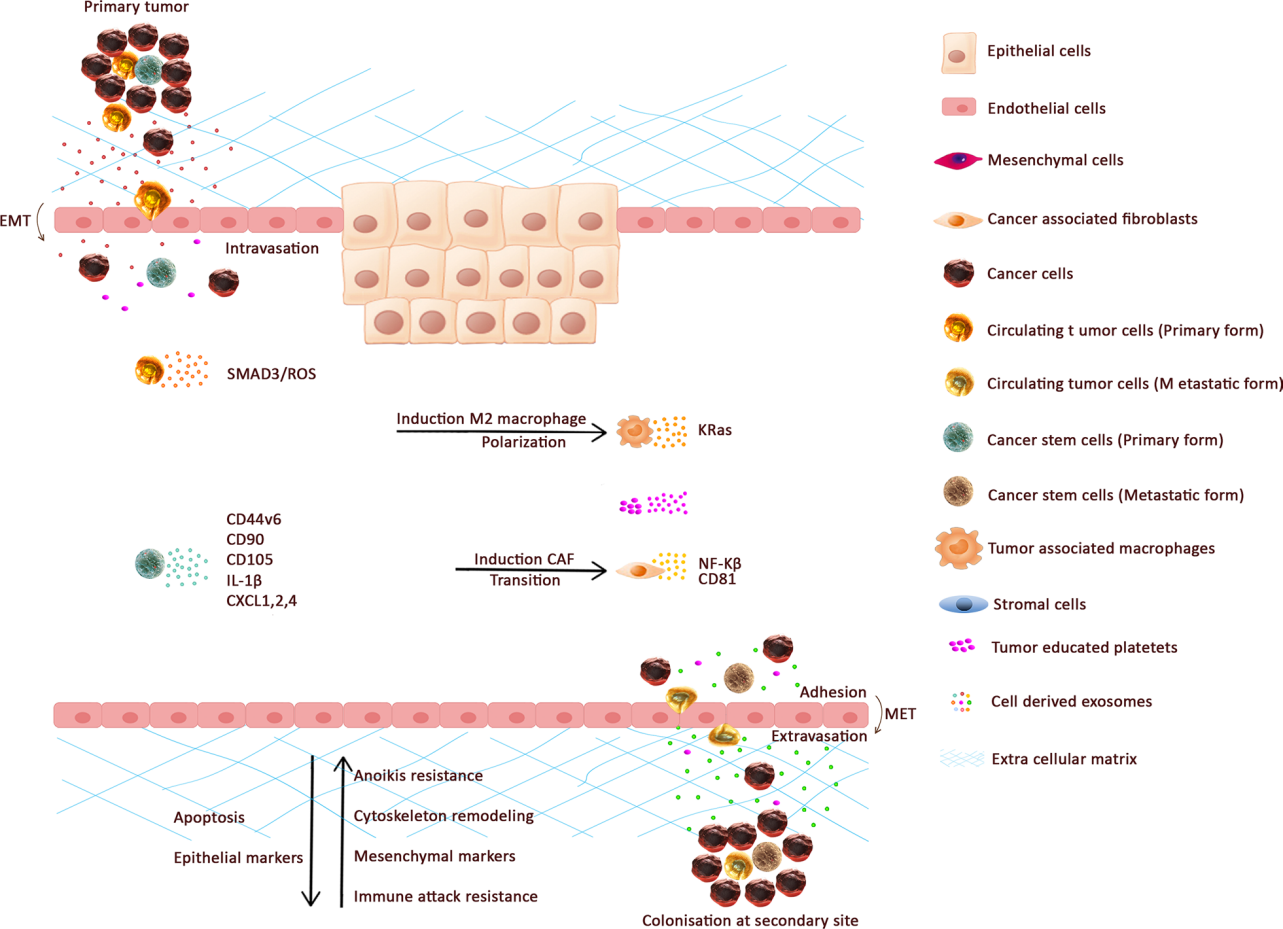
The primary tumor distributes CTCs, CSCs, and TDEs in the CRC microenvironment to metastasize and establish secondary tumors in other organs of body via the blood. Exosome derived CTCs release SMAD3, Exosome derived CSCs release CD44v6, CD90, CD105, IL-1B, CXCL 1, 2, 4. In addition, Exosome derived cancer-associated fibroblast released Nf-KB and CD81

Metabolic cells reprogramming, loss of cell connection with overexpression of matrix metalloproteinases (*MMP*), cancer cells diapedesis and its integration to define target sites contribute in metastasis cascade. Tumor microenvironment (TME) consists of CAFs, extracellular matrix (ECM), cancer- tumor-associated vasculature and inflammatory immune cells. Mediating the crosstalk between tumor and tumor-associated cells identify as a viable step in cancer development (Fig. 3) [126, 127].

Primary TDE conveys messages to the other cells which exist in TME, as well as modifying the microenvironment through their cargo. Not only does TDE play a pivotal role, but also the exosomes secreted by cancer-associated factors including CAFs, tumor-associated macrophages (TAMs), endothelium, leukocytes and progenitor cells should be considered as significant characteristics in cancer progression [128]. TDE is also important in the regulation of macrophage polarization and CAF transition [129].

The data related to the TDE roles in CRC are limited but it was approved that TDE in other cancers promotes invasiveness by regulating signaling pathway, for example, primary TDEs enhance *SMAD3/ROS* signaling and induce CTC survival and cell adhesion. Furthermore, the levels of TDEs markers which participated in EMT process cellular movement and cell–cell signaling in cancer patients’ blood correlated with the disease stage [3]. MiRNAs encapsulated in EVs play a significant role in metastasis such as circulating exosomal *microRNA*-*203* via inducing TAM in CRC [130], [130]. Cha et al. showed that the *KRAS* status of CRC have a direct influence on the type of miRNAs enriched in exosomes [131]. Conditioned media harvested from M2 macrophages which consist of derived exosomes promote CRC motility and invasion throughout *IL6*, *Wnt5a*, *TNFα* and *EGF* molecules [132].

Interestingly, an acidic and hypoxic microenvironment stimulates the release of TDE and is involved in epithelial adheres junctions and cytoskeleton remodeling pathways [133]. In addition, TDEs may potentially collaborate in the dynamic regulation of the tumor fate and is considered as a valuable diagnostic non-invasive approach [34, 134].

## Cancer stem cells regulate tumor microenvironment via exosomes

CSCs or “tumor-initiating cells”, a rare subpopulation are capable of self-renewal and differentiate into specialized cells through symmetric division and therapeutic resistance drive tumor growth [135]. Nowadays, CSCs are investigated in various ranges of solid tumors. CSCs derived EVs contribute in tumor initiation, progression, angiogenesis, invasion and metastasis formation [136].

Tumor exosome RNAs induce the expression of interleukin-1β through NF-κB signaling leading to the survival of neutrophil sustain. Colorectal CSCs secreted *CXCL1* and *2* and attracted neutrophils primed via IL-1β to promote CRC cells tumorigenesis [137]. Moreover, exosomes may transfer mutant *KRAS* to recipient cells and trigger increases in *IL*-*8* production, neutrophil recruitment as well as the formation of the neutrophil extracellular trap (NET), leading to the deterioration of CRC [138]. *CD44v6* CSC-derived exosomes contribute to cancer development by non-cancer initiating cells to acquire the CSC phenotype [139].

EVs-derived CSCs with variable patterns of miRNA can convey their oncogenic features in order to affect cancer proliferation, progression, invasion, metastasis [140], activate angiogenesis and stimulate tumor immune escape mechanisms [141, 142] (Fig. 3).

## Conclusion

Tumor metastasis is still the main principle of cancer death, highlighting the importance of investigating an updating approach to control it. Cross talks among tumor cells and derived-exosomes play a significant role in a dynamic network of cancer microenvironment. Therefore, their recognition and characterization are a crucial step in accurate comprehension of molecular and cellular oncology. Tracking cancer related markers in body fluid could be helpful to measure residual disease presence, recurrence, relapse and resistance and address the needs of clinicians and patients. Liquid biopsy, including CTCs and TDEs as a noninvasive tool in the field of precision medicine, provides substantially helpful information regarding diagnosis, prognosis, predictive and pharmacodynamics.

**Fig. 4:**
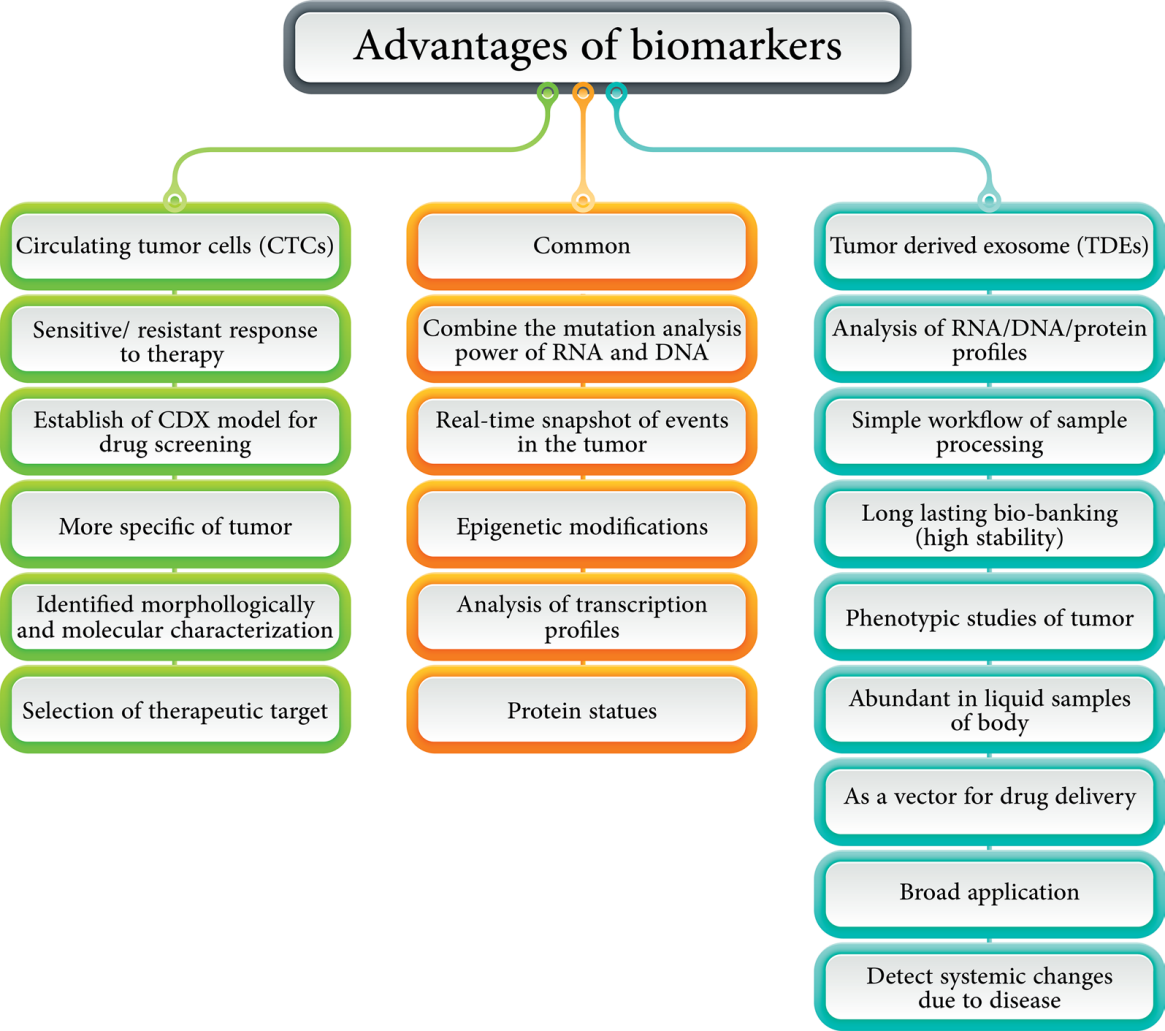
Comparison of the merits of CTCs (green boxes) and TDEs (blue boxes) together. All of the common characterizations of both were drawn in the middle (orange boxes)

In spite of numerous merits that can be counted for CTCs and TDEs separately or simultaneously (Fig. 4), it should be noted that the most challengeable and disadvantageous of them concern isolation and purification due to methodological restrictions (sensitivity and specificity) and standardization because heterogeneity must be resolved. For example, by inducing the apoptosis of CTCs by intervening ROS-mediated DNA damage can inhibit the CTCs metastasis along the the EGF pathway which is cleared by ingenuity exosome pathway analysis [143]. In another study, it was proved that TDEs have equivalent prognostic values to CTCs in the investigated metastatic cancers. Patients with favorable CTC counts can have further prognostic stratification using TDEs [144].

Lab on chip (LOC) technology, in order to grow awareness about the point-of-care testing in cancer was developed and because of low consumption of a sample and high compatibility with the liquid biopsy concept and personalized medicine it has been welcomed [145, 146]. This precious dream can come true with the analysis of patient-activated social networks and systems medicine. P4 medicine that is predictive, personalized, preventive, and participatory can be helpful in this field, next to gene-panel testing due to next-generation sequencing (NGS) technology [147] and plays a critical role in covering the current shortcomings of liquid biopsy regarding practicality, standardization, and the result comparisons.

Despite many techniques regarding CTC exosome capturing and subgrouping are available in clinics; the need for optimization of downstream analysis is tangible. Additionally, distinguishing between CTCs with high and low metastatic status as well as between TDEs and normal status is absolutely vital. In conclusion, liquid biopsy is an expanding field in the management of CRC patient in different stages. It is highly recommended that further research be done on CTCs and TDEs alone or simultaneously until both can serve as valuable biomarkers in clinics.

## Data Availability

Data sharing is not applicable to this article as no new data were created or analyzed in this study and openly available in [repository name at http://doi.org/[doi] and reference number.

## Abbreviations

CRC: Colorectal cancer
CTCs: Circulating tumor cells
TDEs: Tumor-derived exosomes
CT: Computed tomography
MRI: Magnetic resonance imaging
TEPs: Tumor-educated platelets
LOC: Lab-on-a-chip
NGS: Next-generation sequencing
CSCs: Cancer stem cells
ECM: Extracellular matrix
EMT: Epithelial mesenchymal transition
MET: Mesenchymal epithelial transition
OS: Overall survival
PFS: Progression-free survival
EVs: Extracellular vesicles
MVBs: Multivesicular bodies
CK: Cytokeratin
EPCAM: Epithelial cell adhesion molecule
DGC: Gradient density centrifugation
UC: Ultracentrifugation
SEC: Size exclusion chromatography
PEG: Poly ethylene glycol
SEM: Scanning electron microscope
TEM: Transmission electron microscopy
NTA: Nanoparticle tracking assay
DLS: Dynamic light scattering
PDMS: Poly dimethyl siloxane
FAP: Familial adenomatous polyposis
APC: Adenomatous polyposis coli
DCLK1: Doublecortin like kinase 1
LGR5: Leucine-rich repeat-containing G protein-coupled receptor 5
IGF-IR: the insulin-like growth factor 1 receptor
IL-4: Interleukin-4
BMP-4: Bone morphogenetic protein 4
Hsp70: Heat shock proteins
PDXs: Patient-derived xenografts
CDXs: CTC-derived xenografts
TSAP6: Tumor suppressor-activated pathway 6
CAFs: Carcinoma-associated fibroblasts
DKK4: Dickkopf-related protein 4
TAAs: tumor-associated antigens
MMP: Matrix metalloproteinases
TME: Tumor microenvironment
Mef2c: Myocyte enhancer factor 2c
HCC: Hepatocellular carcinoma
HDGF: Hepatoma-derived growth factor
GSCs: Glioma stem cells
CLIC1: contain functionally active Cl-intracellular channel 1
NET: Neutrophil extracellular trap
EGFR: Epidermal growth factor receptor
DEP-FFF: Dielectrophoretic field-flow fractionation
VEGF: Vascular endothelial growth factor
